# Editorial: Tumor microenvironment, immunotherapy, and drug resistance in breast and gastrointestinal cancer

**DOI:** 10.3389/fimmu.2023.1265704

**Published:** 2023-08-29

**Authors:** Xinpei Deng, Jindong Xie, Lu Yang, Dong-Hua Yang, Shaoquan Zheng

**Affiliations:** ^1^ Sun Yat-sen University Cancer Center, State Key Laboratory of Oncology in South China, Guangzhou, China; ^2^ Department of Radiotherapy, Guangdong Provincial People’s Hospital, Guangzhou, China; ^3^ New York College of Traditional Chinese Medicine, New York, NY, United States; ^4^ Department of Breast Surgery, Breast Disease Center, The First Affiliated Hospital, Sun Yat-sen University, Guangzhou, China

**Keywords:** tumor microenvironment, immunotherapy, drug resistance, breast cancer, gastrointestinal cancer

Breast cancer (BC) and gastrointestinal (GI) cancers have historically been recognized for their significant heterogeneity and challenging nature, resulting in elevated rates of morbidity and mortality (1). Despite notable advancements and clinical achievements in cancer immunotherapies, particularly immune checkpoint blockade (ICB) therapy, which has demonstrated efficacy in various solid tumors such as melanoma (2), its effectiveness in patients with BC and GI cancers remains unsatisfactory (3, 4). The tumor microenvironment (TME), comprising non-malignant host cells and non-cellular constituents within the tumor, exerts a pivotal impact on tumorigenesis, progression, metastasis, and therapeutic resistance *via* dynamic interactions with neoplastic cells (5). The regulation of the tumor immune microenvironment, which is a notable tumor-extrinsic factor, plays a pivotal role in the intricate mechanisms of drug resistance observed in breast and gastrointestinal cancers, particularly in the context of immunotherapy. Despite the significant advancements made in TME-targeted cancer therapy, such as ICB, the clinical efficacy of therapeutic approaches targeting the TME still falls short of expectations. Accordingly, there is a pressing need to ascertain novel targets and biomarkers that have the potential to enhance clinical effectiveness and precision in distinct patient populations. This Research Topic, titled “*Tumor Microenvironment, Immunotherapy, and Drug Resistance in Breast and Gastrointestinal Cancer*,” was curated by 3 guest editors and comprises a collection of 9 articles, including 3 reviews and 6 original research studies. These articles offer a profound comprehension and novel comprehensive perspective on the tumor microenvironment, immunotherapy, drug resistance mechanisms, and potential strategies for overcoming these challenges in breast and gastrointestinal cancers.

BC ranks among the malignancies with the highest morbidity rates globally, primarily due to its increasing incidence. In addition to conventional treatment approaches, immunotherapy, specifically ICB, has significantly revolutionized the treatment paradigm for BC patients, particularly those diagnosed with triple negative breast cancer (TNBC). However, it is important to note that only a subset of patients can derive therapeutic benefits from this modality. In order to facilitate future advancements in the field of immunotherapy resistance in TNBC, Zheng et al. systemically combed the publication trends and research highlights of response of immunotherapy in TNBC treatment. The authors presented a comprehensive synthesis of the various mechanisms of immune evasion observed in TNBC, organizing them into three distinct categories: loss of tumor-specific antigen, deficiency in antigen presentation, and inability to initiate an immune response. Additionally, they examined the aberrant activation of various immune-critical signaling pathways. Subsequently, they investigated the cumulative influence of these actions on molding the immunosuppressive milieu within the TME, thereby elucidating the potential molecular mechanism and targets associaed with resistance to immunotherapy in TNBC. To find out the signature factors reflecting the TME and therapeutic response, Li et al. devised a prognostic signature utilizing 27 genes associated with DNA damage repair. This signature was employed to evaluate the prognosis, tumor immunometabolic profile, and therapeutic response in cases of BC. The authors effectively showcased the efficacy of these signatures in prognosticating patient outcomes, immunometabolic profiles, and therapeutic sensitivity. These findings possess the capacity to propel precision medicine forward and streamline the discovery of novel therapeutic targets for BC. Furthermore, Li and Liu presented a comprehensive analysis of the involvement of natural killer (NK) cells and antibody-dependent cellular cytotoxicity (ADCC) in the targeted treatment of HER2-positive BC. Subsequently, they examined the regulatory mechanisms and recent advancements in utilizing NK cells and ADCC as an immunotherapeutic approach for HER2-positive BC.

Novel prognostic biomarkers for the diagnosis and prognosis of BC, as well as the signature of the tumor microenvironment, were also investigated. Yang et al. introduced the methods used to detect peripheral blood autoantibodies and the research progress in the screening and prognosis of BC made in recent years to provide a potential direction for the examination and treatment of BC. Pei et al. constructed a prognostic model based on public databases, which incorporates single-cell sequencing analysis, weighted co-expression network analysis, and transcriptomic differential expression analysis. This model based on multi-discipline techniques sheds light on exploiting robust predicting tools to promote efficacies of immunotherapy in BC. On the other hand, Luo et al. constructed a degradome-related (DR) prognostic signature that correlates with immune infiltration and tumor mutation burden based on differentially expressed DR genes set from The Cancer Genome Atlas (TCGA), METABRIC and GSE96058 cohorts. They identified 10 genes in predicting prognosis, risk stratification and guiding treatment for patients with BC.

This Research Topic has also contributed to a profound comprehension and novel comprehensive perspective on TME, immunotherapy and drug resistance in GI cancers. Gastric cancer (GC) is a prevalent malignancy affecting the gastrointestinal tract, exhibiting a high global prevalence, notable incidence and mortality rates, and an unfavorable prognosis (1). Although immunotherapy has gained approval for the treatment of advanced GC, the median overall survival time remains notably brief (6). Li et al. conducted a bioinformatic analysis utilizing single-cell RNA sequencing on ten GC specimens, both before and after treatment with neoadjuvant camrelizumab plus mFOLFOX6. The findings of this study demonstrate the interplay between the TME and the efficacy of neoadjuvant immunotherapy combined with chemotherapy in GC. Additionally, the analysis underscores the significance of CD8+ T cells as a predictive marker for response to this combination therapy in GC. Colorectal cancer with proficient mismatch repair/microsatellite stability status continues to exhibit limited response to immunotherapies, despite the significant advancements achieved by this treatment modality in other types of gastrointestinal cancers (7). Hu et al. performed an exploratory analysis wherein they employed RNA sequencing to examine primary colorectal lesions, colorectal liver metastatic lesions, and normal liver tissues. Their findings indicate that histopathological growth patterns are discernible morphological alterations that arise from the regulation of molecular expressions. This regulation is a cumulative consequence of the heterogeneity and remodeling of primary tumor seeds and liver soils. Meanwhile, Wang et al. elucidated the immunological and prognostic biomarker of NCAPG2 in various types of cancer, with a specific focus on its validation in pancreatic cancer. Nevertheless, further investigations are warranted to ascertain its suitability for clinical applications.

In summary, this Research Topic encompasses a comprehensive examination and analysis of 9 articles that delve into the topics of TME, immunotherapy, and drug resistance mechanisms in the context of BC and GI cancers. These articles approach the subject matter from various perspectives, offering insights and potential strategies to enhance treatment efficacy. Regrettably, the Research Topic does not encompass the advancements made in immunotherapy for esophageal cancer and hepatocellular carcinoma, which warrants further investigation. TME and immunotherapy has the potential to synergistically generate a potent anti-tumor response, even in cases where tumors have exhibited resistance to ICB therapy. This innovative concept offers a promising avenue for future research and clinical application, with the potential to enhance treatment outcomes and patient prognosis in the context of BC and GI cancer. It is our aspiration that the discoveries presented in this Research Topic will serve as a catalyst for future innovations, fostering an exciting and promising future in this field.

## Author contributions

XD: Writing – original draft. JX: Writing – original draft. LY: Writing – review & editing, Supervision. D-HY: Writing – review & editing, Conceptualization. SZ: Writing – review & editing, Conceptualization.
